# Symptoms associated with healthcare resource utilization in the setting of inflammatory bowel disease

**DOI:** 10.1038/s41598-022-14838-y

**Published:** 2022-06-22

**Authors:** Kaleb Bogale, Parth Maheshwari, Mitchell Kang, Venkata Subhash Gorrepati, Shannon Dalessio, Vonn Walter, August Stuart, Walter Koltun, Nana Bernasko, Andrew Tinsley, Emmanuelle D. Williams, Kofi Clarke, Matthew D. Coates

**Affiliations:** 1 Department of Medicine, Pennsylvania State University College of Medicine, Hershey, PA USA; 2 Department of Medicine, Division of Gastroenterology and Hepatology, Pennsylvania State University College of Medicine, 500 University Drive, M.C. HU33, Hershey, PA 17033 USA; 3 Department of Public Health Sciences, Pennsylvania State University College of Medicine, Hershey, PA USA; 4 Department of Surgery, Division of Colorectal Surgery, Pennsylvania State University College of Medicine, Hershey, PA USA; 5 Department of Pharmacology, Pennsylvania State University College of Medicine, Hershey, PA USA

**Keywords:** Inflammatory bowel disease, Health care

## Abstract

Several symptoms have been connected to increased healthcare resource utilization (HRU) in the context of inflammatory bowel disease (IBD), including both Crohn’s disease (CD) and ulcerative colitis (UC). This study was designed to investigate the prevalence of IBD-associated symptoms and to determine whether any are independently associated with HRU. We undertook a retrospective analysis of data related to consecutive IBD patient encounters from a tertiary care referral center between 1/1/2015 and 8/31/2019. Demographics, clinical activity, endoscopic severity, IBD-related symptom scores, anxiety and depression scores, and other key clinical data were abstracted. Four hundred sixty-seven IBD patients [247f.: 220 m; 315 CD, 142 UC and 11 indeterminate colitis] were included in this study. The most common symptoms were fatigue (83.6%), fecal urgency (68.2%) and abdominal pain (63.5%). Fatigue, abdominal pain, anxiety or depression, corticosteroids, and opioids were each positively associated with HRU, while NSAID and mesalamine use were inversely associated on bivariate analysis. The only factor that demonstrated a statistically significant association with HRU in the whole cohort on multivariable analysis was abdominal pain. Abdominal pain is independently associated with HRU and should be specifically screened for in IBD patients to identify individuals at risk of undergoing expensive interventions. This study also reinforces the importance of optimizing diagnostic and therapeutic management of abdominal pain in IBD.

## Introduction

Inflammatory bowel disease (IBD), including Crohn’s disease (CD) and ulcerative colitis (UC), are chronic disorders of the gastrointestinal tract that cause inflammation within the gut, extra-intestinal manifestations (EIMs), and a myriad of symptoms. IBD has demonstrated a progressively increasing global incidence and approximately 3 million individuals are estimated to have IBD in the United States (U.S.) alone. Management of IBD represents a significant public health challenge^1,2^. Yearly, patients with IBD incur significant direct medical costs, projected at an average of $22,987 per patient in the U.S., and indirect costs due to lost earning potential and sick leave^3–5^. Importantly, healthcare costs associated with IBD are increasing yearly, with an estimated 7% (CD) and 10% (UC) increase per year from 2006 through 2016^6–9^. However, there also appears to be a relative minority of patients driving the majority of healthcare costs in IBD. These patients are typically afflicted with more severe disease activity and demonstrate a higher likelihood of comorbid anxiety, depression, and chronic pain disorders^10,11^. In this setting, there is a growing need to identify economical strategies to reduce healthcare resource utilization (HRU) and overall costs of IBD management.

Numerous clinical and demographic factors have previously been associated with HRU in patients with IBD. Historically, gut-related inflammation and disease complications (including strictures, fistulae, and abscesses) have been considered the primary drivers of healthcare resource utilization^12,13^. Over time, additional disease characteristics (e.g., disease extent and location, EIMs), co-morbidities (psychiatric illness), low socio-economic status, and the use of certain medications (e.g., corticosteroids, narcotics, and biologic therapy) have also been associated with HRU^6,14,15^. Additionally, patients undergoing psychotherapy for depression were less likely to exhibit HRU^16^. Despite these observations, previous studies on this topic are sometimes contradictory to one another. Even when considering more consistently exhibited predictors of HRU (e.g., active inflammation, disease type, disease extent, disease complications), it can be difficult for providers to routinely and reliably assess those parameters. Therefore, there is an ongoing search to identify more efficient and cost-effective methods to predict risk for HRU in patients with IBD.

It is possible that basic IBD symptom assessments could fulfill this need. Patients with IBD can experience a wide array of symptoms, including diarrhea, fatigue, abdominal pain, nausea or vomiting, weight loss, bloody stools, and fecal urgency^6,17–20^. These symptoms are relatively easy to identify, cheap to screen for and routinely assessed in this patient population. This is particularly relevant as large-scale studies of digestive disorders have implicated specific symptoms (e.g., abdominal pain) as major drivers of cost and HRU in general^21^. In fact, a recent study has linked at least one symptom (e.g., abdominal pain) with HRU in IBD^11^.

To date, no study has undertaken a comprehensive assessment of IBD-related symptoms, particularly in the context of other previously identified predictors of HRU, to examine their potential relationship to resource use in this patient population. Given the potential value in using symptom screening to identify patients at risk of HRU, we performed this study to evaluate for potential relationships between common IBD-related symptoms and HRU, while simultaneously considering other key factors previously linked to HRU in IBD (e.g., disease activity, disease complications, medication use).

## Methods

### Study population and selection criteria

We performed a retrospective analysis of data recorded in relation to consecutively evaluated patients at our institution between 1/1/2015 and 8/31/2019. The data for this study includes clinical and research information related to healthcare encounters for patients treated within the dedicated IBD center at PSHMC, a tertiary care referral hospital. All participants in this study were > 17 years old and had an established diagnosis of CD, UC, or IBD colitis of indeterminate nature, based upon standard clinical criteria routinely used to identify IBD. Additionally, all participants had to have completed surveys on IBD-related symptoms including the Harvey-Bradshaw Index (HBI)^22^, Simple Clinical Colitis Activity Index (SCCAI)^23^, Hospital Anxiety and Depression Scale (HADS)^24^, Short Inflammatory Bowel Disease Questionnaire (SIBDQ)^25^ and healthcare utilization and undergone a contemporaneous ileocolonoscopy (e.g., within one month of survey completion). We excluded any individuals with active malignancy, pregnancy, perianal disease as well as UC patients who had undergone colectomies. Informed consent was obtained from all participants. This work was performed in accordance with the ethical standards defined in the 1964 Declaration of Helsinki and its later amendments. Penn State College of Medicine Institutional Review Board approved this study under protocol STUDY00013788.

### Definitions and data abstraction

Healthcare resource utilization (HRU) was defined as recent, patient-reported participation in an IBD-related imaging study, emergency room visit, hospitalization and/or surgery based upon responses to a survey administered during our clinical encounter with the patient. Disease activity was based upon ileocolonoscopic evaluation. Disease activity was assessed in CD with the Simple Endoscopic Score for CD (SES-CD), which ranges from 0 to 2 (remission), 3 to 6 (mild endoscopic activity), 7 to 15 (moderate endoscopic activity), and greater than 15 (severe endoscopic activity) (in accordance with the approach utilized by several previous studies)^26^. Thus, moderate to severe disease activity in CD was defined as a SES-CD greater than or equal to 7 in any one intestinal segment. Disease activity was assessed in UC with the Mayo endoscopy sub-score, which ranges from 0 (no disease) to 3 (severe disease). Thus, moderate to severe disease activity in UC was defined as a Mayo endoscopy sub-score of 2 or 3.

Prior to each ileocolonoscopy, patients completed surveys that included questions specifically relating to IBD-related symptoms. Abdominal pain was screened through two separate items: (1) the fourth question in the SIBDQ (“How often over the past two weeks have you experienced abdominal pain?”, where patients respond using a frequency-based inverse Likert scale, with 1 representing pain “all of the time” and 7 representing pain “none of the time”), and (2) the second question from the HBI, which included potential responses of 0 (“no abdominal pain”), 1 (“mild”), 2 (“moderate”) and 3 (“severe”). Thus, we defined clinically relevant abdominal pain as a numeric rating less than or equal to 5 on the SIBDQ pain score or greater than or equal to 1 on the HBI pain score. Presence of anxiety or depression symptoms were determined based upon responses to the Hospital Anxiety and Depression Scale (HADS) completed at the time of the clinical encounter, which ranges from 0 to 7 (normal), 8 to 10 (borderline abnormal), and 11 to 21 (abnormal). HADS anxiety or depression sub-scores of 8 or greater was determined as clinically significant presence of anxiety or depression. A comprehensive review of additional symptoms was determined through totals and sub-scores of the HBI, SCCAI, and SIBDQ surveys. The symptoms specifically evaluated for in these surveys were: fatigue, diarrhea, rectal bleeding, fecal urgency, tenesmus, gas, and extra-intestinal manifestations (EIMs), including inflammatory arthritides/arthralgias, pyoderma gangrenosum, erythema nodosum, uveitis, episcleritis, and primary sclerosing cholangitis. We evaluated for the current presence of each of these symptoms at the time that the surveys were completed. Reported experience with these symptoms on any of the surveys above was used to indicate their presence (with diarrhea being defined as greater than three bowel movements per day).

Additional demographic and clinical characteristics were abstracted from the medical record, including patient age, sex, disease complications (defined in this study as previous or current gastrointestinal stricture, intra-abdominal fistula, and/or abscess), surgical history, current medications (including mesalamine, immunomodulator, biologic, antidepressant/anxiolytic, corticosteroid, opioid, and NSAID usage), and tobacco use.

### Statistical analysis

The primary outcome of interest was healthcare resource utilization (HRU) (as defined above) in the complete IBD cohort (n = 467) and in each of the subtypes of IBD (CD and UC). Secondary outcomes of interest were prevalence of symptoms. Data were extracted and analyzed using GraphPad Prism version 8 (San Diego, CA) or SAS version 9.4 (Cary, NC). We performed descriptive statistics and bivariate analyses (e.g., Student’s t-test for continuous variables and Chi-square or Fisher’s exact test for categorical variables as appropriate) comparing demographic and clinical factors (including the presence of each symptom described above) in two cohorts: 1) IBD patients demonstrating HRU and 2) IBD patients with no HRU. A multivariable logistic regression model was then created which incorporated key clinical factors associated with HRU in prior studies or found to be significantly (*p* < 0.05) or near significantly (*p* = 0.05–0.1) associated with HRU in our bivariate analysis. Odds ratios (ORs) and corresponding 95% confidence intervals (CIs) are reported from the logistic regression models. *P* values of < 0.05 were considered to be statistically significant.

## Results

### Study participants

Four hundred sixty-seven consecutively enrolled patients with IBD (247 females, 220 males) were included. Of the study participants, 315 were diagnosed with CD, 142 with UC, and 10 with indeterminate colitis (Table 1). The mean age was 44 years, ranging from 19 to 90 years. On endoscopic evaluation, 174 (37.1%) demonstrated moderate to severe disease activity. One hundred ninety-two individuals (40.9%) had disease complications (i.e., previous or current gastrointestinal stricture, intra-abdominal fistula, and/or abscess) and 151 (32.2%) had extra-intestinal manifestations (Table 1). In the total study cohort, 225 patients (48.0%) used biologic therapies, 123 patients (26.2%) used immunomodulatory therapies, and 99 (21.1%) were treated with mesalamine. Our study cohort reported tobacco use in 9.4%, opioid use in 11.1%, and NSAID use in 18.1% (Table 1). Participants with UC (45.8%) had a statistically significant increased frequency of moderate to severe endoscopically-confirmed active disease than participants with CD (33.0%) (p < 0.01).

**Table 1:** Clinical Characteristics of Healthcare Resource Users and Non-Users.

	Total (n = 467)	HRU (n = 305)	No HRU (n = 163)	Odds Ratio	95% Confidence Interval		*P* Value
**Demographics and disease characteristics**							
Age (mean years ± SEM)	44.0 ± 0.7	43.7 ± 0.9	44.7 ± 1.3				0.493
Gender [female (%)]	247 (52.7%)	160 (52.5%)	87 (53.4%)	1.03	0.70	1.51	0.878
IBD Subtype (CD/UC/IC)	315/142/11	211 / 87 / 7	104 / 55 / 4	1.28	0.85	1.94	0.236
Moderate to Severe inflammation (%) (on endoscopic evaluation)	174 (37.1%)	115 (37.7%)	59 (36.2%)	1.08	0.73	1.60	0.708
Disease complication (%)	192 (40.9%)	134 (43.9%)	58 (35.6%)	1.42	0.96	2.10	0.081
**Medication use**							
Biologic use (%)	225 (48.0%)	154 (50.5%)	71 (43.6%)	1.32	0.90	1.94	0.153
Mesalamine use (%)	99 (21.1%)	51 (16.7%)	48 (29.4%)	0.48	0.31	0.76	0.001
Immunomodulator use (%)	123 (26.2%)	82 (26.9%)	41 (25.2%)	1.09	0.71	1.69	0.685
Corticosteroid use (%)	67 (14.3%)	52 (17.0%)	15 (9.2%)	2.03	1.10	3.73	0.023
Antidepressant or Anxiolytic (%)	119 (25.4%)	80 (26.2%)	39 (23.9%)	1.13	0.73	1.76	0.586
NSAID use (%)	85 (18.1%)	47 (15.4%)	38 (23.3%)	0.60	0.37	0.97	0.036
Opioid use (%)	52 (11.1%)	41 (13.4%)	11 (6.7%)	2.15	1.07	4.30	0.031
Tobacco use (%)	44 (9.4%)	35 (11.5%)	9 (5.5%)	2.10	0.97	4.55	0.061
**Symptoms**							
Extra-intestinal manifestations (%)	151 (32.2%)	104 (34.1%)	47 (28.8%)	1.28	0.85	1.95	0.239
Arthralgia (%)	117 (24.9%)	84 (27.5%)	33 (20.2%)	1.50	0.95	2.37	0.084
Dermatopathies (%)	41 (8.7%)	29 (9.5%)	12 (7.4%)	1.35	0.67	2.72	0.405
Anxiety or Depression (%)	236 (50.3%)	166 (54.4%)	70 (42.9%)	1.59	1.08	2.33	0.018
Fatigue (%)	392 (83.6%)	266 (87.2%)	126 (77.3%)	2.00	1.22	3.29	0.006
Fecal urgency (%)	320 (68.2%)	212 (69.5%)	108 (66.3%)	1.16	0.77	1.74	0.471
Abdominal pain (%)	298 (63.5%)	216 (70.8%)	82 (50.3%)	2.40	1.62	3.55	< 0.001
Tenesmus (%)	228 (48.6%)	155 (50.8%)	73 (44.8%)	1.27	0.87	1.87	0.214
Gas (%)	220 (46.9%)	147 (48.2%)	73 (44.8%)	1.15	0.78	1.68	0.481
Diarrhea (%)	182 (38.8%)	118 (38.7%)	64 (39.3%)	0.98	0.66	1.44	0.903
Rectal bleeding (%)	170 (36.2%)	117 (38.4%)	53 (32.5%)	1.29	0.87	1.93	0.211

HRU = Healthcare Resource Utilization, CD = Crohn’s disease, UC = ulcerative colitis, IC = indeterminate colitis. SEM = standard error measurement. Disease Complication = intra-abdominal stricturing, fistulae and/or abscess. IBD subtype analysis reports OR and CIs for CD vs. UC (excluding patients with IC).

### Prevalence of IBD-related symptoms

**Figure 1:**
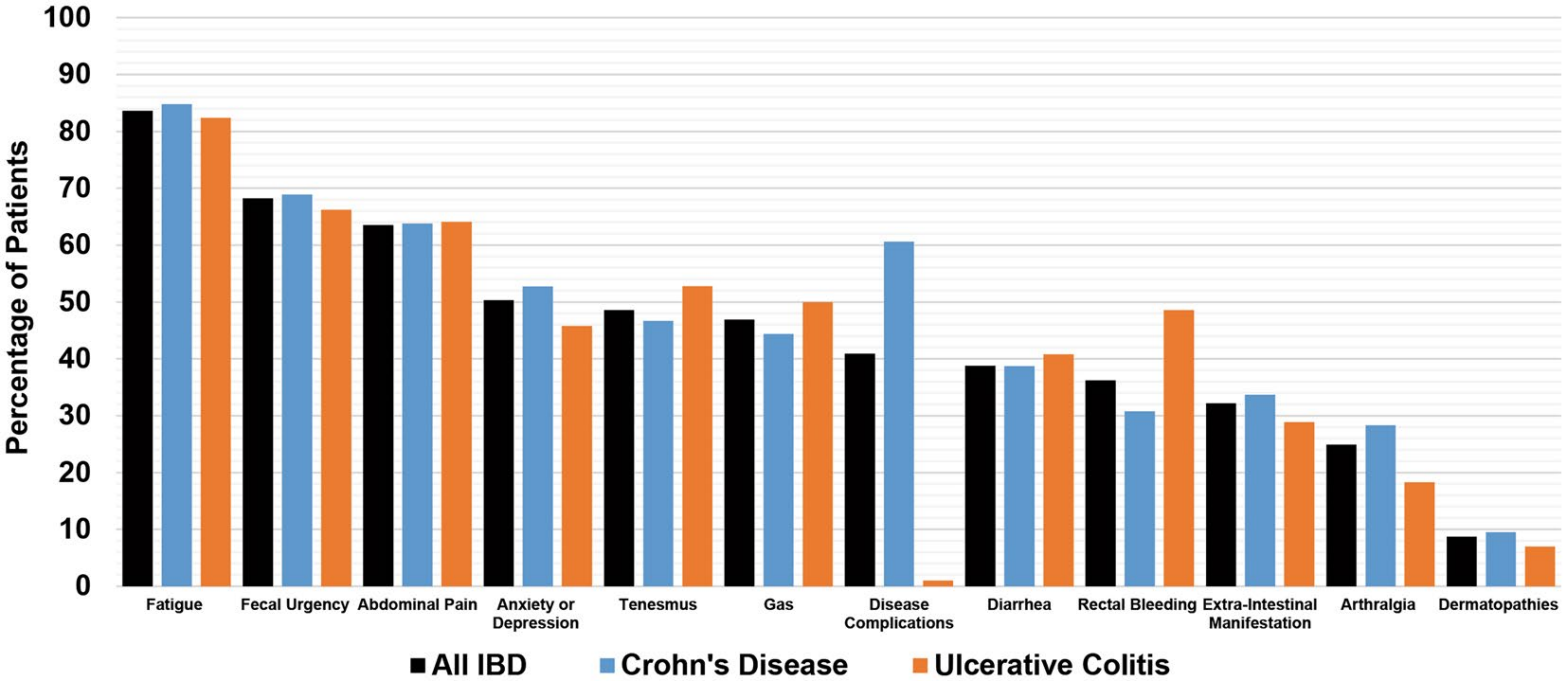
Symptom/Complication Prevalence in Inflammatory Bowel Disease.

Figure 1 displays the prevalence of IBD-related symptoms surveyed for in the study population. The most common symptoms were fatigue (83.6%), fecal urgency (68.2%), abdominal pain (63.5%), tenesmus (48.6%), gas (46.9%), anxiety or depression (50.3%), and diarrhea (38.8%). There were similar rates of symptoms between CD and UC, except an increased frequency of rectal bleeding in UC [CD (30.6%) and UC (48.6%), *p* < 0.01].

### Healthcare resource utilization in IBD

Of the 467 study participants, 305 (65.3%) demonstrated HRU (i.e., participation in an IBD-related imaging study, emergency room visit, hospitalization or surgery). IBD healthcare resource utilizers were more likely to demonstrate fatigue (*p* < 0.01), anxiety or depression (*p* = 0.02), and abdominal pain (*p* < 0.01) (Table 1). There were no statistically significant differences in the remaining symptoms reported when considering HRU (Table 1). HRU in IBD was more likely to be associated with corticosteroid use (*p* = 0.02) and opioid use (*p* = 0.03), while NSAID use (*p* = 0.04) and mesalamine use (*p* < 0.01) were less likely associated with HRU in IBD (Table 1). There were no other statistically significant differences in the prevalence of disease subtypes (CD or UC), presence of moderate to severe disease activity, age, other IBD medication, tobacco use, or sex when considering HRU (Table 1).

Table 2 demonstrates results from our multivariable model which includes clinical factors previously associated with HRU (stricturing and fistulizing disease^6,12,13^, anxiety or depression^12,15^, and steroid or opioid use^15^) and clinical characteristics with a statistically significant (*p* < 0.05) or near statistically significant (*p* = 0.05–0.10) association with HRU in our bivariate analysis. This included IBD-related symptoms (including fatigue, anxiety or depression, and arthralgias [*p* = 0.084 in bivariate analysis]), NSAID use, and mesalamine use. Of note, all 467 study participants were included in the multivariable logistic regression model. Abdominal pain (OR 1.86, 95% CI 1.19–2.92, *p* < 0.01) was the only factor statistically associated with HRU on multivariable analysis (Table 2), even while simultaneously considering the presence of disease complications, medications (mesalamine, corticosteroids, NSAIDs, or opioids), or other symptoms (including fatigue, anxiety or depression, and arthralgias) (Table 2).

**Table 2:** Multivariable Logistic Regression Model, Healthcare Resource Utilization in IBD.

Variable	Odds ratio	95% Confidence limits		*P* Value
Disease complications	1.28	0.83	1.96	0.265
Anxiety or depression	1.04	0.67	1.61	0.872
Fatigue	1.42	0.80	2.51	0.227
Abdominal pain	**1.86**	**1.19**	**2.92**	**0.007**
Arthralgia	1.09	0.66	1.79	0.730
Steroid use	1.68	0.88	3.20	0.117
NSAID use	0.61	0.37	1.01	0.056
Opioid use	1.65	0.79	3.41	0.180
Mesalamine use	0.62	0.38	1.01	0.057

IBD = inflammatory bowel disease, Disease Complications = intra-abdominal stricturing, fistulae and/or abscess.

### IBD subtype and healthcare utilization

When we focused on the CD cohort (n = 315), the only clinical factors positively associated with HRU on bivariate analysis were abdominal pain (n = 201 [63.8%], OR 2.40, 95% CI 1.48–3.90, *p* < 0.01), corticosteroid use (n = 46 [14.6%], OR 2.24, 95% CI 1.04–4.85, *p* = 0.04), and opioid use (n = 39 [12.4%], OR 3.03, 95% CI 1.23–7.48, *p* = 0.02). There were no statistically significant differences in the prevalence of disease complications or moderate-severe disease activity, extra-intestinal manifestations, age, IBD medication use, or other IBD-related symptoms when considering HRU. A multivariable model was conducted including all variables with a statistically significant (*p* < 0.05) or near significant (*p* = 0.05–0.10) association with HRU, which included IBD-related symptoms of abdominal pain and rectal bleeding (*p* = 0.07 in bivariate analysis), and medications (opioid use and corticosteroid use). Abdominal pain (OR 1.99, 95% CI 1.20–3.21, *p* < 0.01) was the only variable independently associated with HRU (Supplemental Table 1).

When we evaluated the UC cohort (n = 142), the only clinical factors positively associated with HRU on bivariate analysis were fatigue [n = 117 (82.4%), OR 5.56, 95% CI 2.14–14.46, *p* < 0.01], abdominal pain [n = 91 (64.1%), OR 2.22, 95% CI 1.10–4.50, *p* = 0.03], presence of anxiety or depression [n = 65 (45.8%), OR 2.75, 95% CI 1.35–5.60, *p* < 0.01], and biologic use [n = 44 (31.0%), OR 2.44, 95% CI 1.11–5.39, *p* = 0.03], while mesalamine use [n = 62 (43.7%), OR 0.33, 95% CI 0.17–0.67, *p* < 0.01], NSAIDs [n = 32 (22.5%), OR 0.28, 95% CI 0.12–0.64, *p* < 0.01] and age (mean = 44.01 ± 74 years, OR 0.98, 95% CI 0.96–1.00, *p* = 0.02) were inversely associated with HRU. There were no statistically significant differences in the prevalence of moderate-severe disease activity, extra-intestinal manifestations, or other IBD-related symptoms reported in regards to HRU. A multivariable logistic regression analysis was conducted including all variables with a statistically significant (*p* < 0.05) or near significant (*p* = 0.05–0.10) association with HRU, which incorporated age, disease activity (*p* = 0.056 in bivariate analysis), anxiety or depression, IBD-related symptoms of fatigue and abdominal pain, and medications (biologic, mesalamine, and NSAID use). The presence of fatigue (OR 9.48, 95% CI 2.63–34.23, *p* < 0.01) and biologic use (OR 2.62, CI 1.01–6.82, *p* < 0.05) were positively associated with HRU, while NSAID use (OR 0.19, 95% CI 0.07–0.54, *p* < 0.01) was inversely associated (Supplemental Table 2).

## Discussion

This study found that abdominal pain was independently associated with healthcare resource utilization in IBD. When considering disease sub-type, abdominal pain was independently associated with HRU in CD, while fatigue and biologic use were each independently associated with HRU only in UC. This is the first study to consider multiple, major IBD-related symptoms simultaneously with other previously reported drivers of HRU, including a wide variety of demographic and disease characteristics. Considering this fact, it is quite notable that specific symptoms (independent of disease activity and complications) were the only factors independently associated with HRU. This work is also important because it demonstrates that simple, inexpensive and widely available clinical information (i.e., symptoms, including abdominal pain) may be effective tools to identify patients at high risk of incurring expensive interventions.

Several findings from this investigation were similar to those of previous studies. For example, there was frequent HRU among patients with IBD in our study, including in both CD and UC^5^. Numerous previous reports suggest an increasing trend in hospitalizations among patients with IBD in the U.S. (throughout 2005–2016^27^, 1994–2005^28^, 1990–2003^29^, and 1998 to 2004^30^). There are also multiple reports of increased emergency department utilization among patients with IBD in the U.S. (throughout 2005–2016^27^ and 2006–2014^31^), though one report found a relatively stable rate (throughout 2009–2011^32^). Additionally, measures of abdominal pain have also previously been associated with HRU in digestive disorders in general^21^, and in IBD specifically^11^.

Unlike previous investigations, however, our study did not demonstrate a clear link between IBD activity and HRU. This is notable because, in contrast to previous investigations, we utilized concurrent, direct assessments of patient disease activity status (i.e., endoscopic evaluation). Additionally, we found no independent associations between HRU and disease-related complications (i.e., strictures and/or fistulae), opioid use, corticosteroid use, or psychiatric symptoms, unlike some previous studies^12,13,33^. In other words, it was the symptoms and not the disease process that were most closely associated with HRU in this setting. Prior studies have suggested that symptoms in IBD may be frequently driven by factors other than inflammatory activity and/or inflammatory complications. For example, in the case of abdominal pain and/or fatigue in IBD, there is evidence that coexistent psychiatric disorders (ex: anxiety and/or depression)^20,34^, peripheral and/or central sensitization of nociceptive pathways^35^ and/or alterations of the gut-brain axis^36,37^, nutritional deficiencies and anemia^38^, sleep disturbances^39^, changes in the microbiome, and/or persistence of previously unrecognized, “subclinical” levels of inflammation^40^ can each play an important role. Future attempts to evaluate abdominal pain and fatigue in IBD (in the context of HRU or otherwise) should consider these factors.

This study also demonstrated differences between the IBD subtypes. As indicated above, while abdominal pain was independently associated with HRU in the total IBD cohort and CD sub-cohort, it was not when considering the UC sub-cohort. This is notable, as IBD subtype was not statistically associated with HRU on bivariate analysis. It is certainly possible that our analysis was underpowered to evaluate for this type of association, particularly considering the substantially smaller number of UC patients included in the analysis. However, this finding may not be surprising, considering that some previous studies have indicated that abdominal pain has been reported more frequently in CD populations^41,42^. In the case of UC, fatigue and biologic use were independently associated with HRU, while NSAID use was inversely associated with HRU. The associations with fatigue and biologic use are perhaps unsurprising considering how common the symptom of fatigue is in IBD and UC^36,37^ and the fact that use of biologic medication is an indicator of more severe disease. The inverse association with NSAID use is also notable considering that several prior studies have demonstrated direct associations between NSAID use and poor outcomes in the setting of IBD^43,44^, though not necessarily with UC^44^. Each of the associations described above warrant further evaluation in larger cohorts of CD and UC patients.

There were several limitations to this study. First, it was a retrospective investigation and so is at risk of a variety of biases. This includes potential recall bias (ex: inappropriate patient survey responses) and/or selection bias (ex: skewing of the study population as a result of the inclusion and exclusion criteria). The data were also gathered from a single, tertiary academic medical center in the United States. Thus, our findings may not be applicable for all patient populations, including those in other countries. Additionally, while patients were asked about any instance of HRU (and were not limited to those events occurring only at our institution), we were unable to verify potential instances of HRU outside of our medical system. Thus, our assessment may have underestimated HRU in this study cohort. As surgery was incorporated into the definition of HRU, we could not properly evaluate it separately as a potential predictor of HRU. This is relevant because some previous studies have demonstrated that surgery may be independently associated with HRU^13^. We were unable to collect potentially relevant laboratory values in all of the study participants, including hemoglobin, measures of nutrition and inflammatory markers such as the erythrocyte sedimentation rate and C-reactive protein. These values have either previously been associated with HRU or have potential for such an association^11,15,45^. As indicated above, this study may have been underpowered to evaluate for some associations within the IBD sub-cohorts, particularly when considering the small UC group. This was also relevant considering that we did not include individuals with perianal disease. Finally, our study could not incorporate a direct assessment of financial cost for the interventions being evaluated, somewhat limiting our overall understanding of the impact that our measures of HRU were having on the individual and society.

In summary, this study represents one of the first efforts to simultaneously assess IBD-related symptoms and other major clinical and demographic variables when considering healthcare resource utilization in the context of IBD. We demonstrated that abdominal pain is independently associated with HRU in IBD. We also found that abdominal pain was independently associated with HRU in CD, while fatigue and use of biologic medications were each independently associated with HRU in UC. The findings of this investigation suggest that screening for these symptoms, along with certain key historical elements (e.g., use of biologic medication), may provide simple and cost-effective means to rapidly identify and risk-stratify IBD patients at risk of undergoing expensive medical interventions. It also reinforces the importance of optimizing management of abdominal pain and fatigue in IBD. We recommend that guidelines for management of IBD should include regular screening for these symptoms in order to help identify patients at risk for costly medical interventions. It is also vital that further research into the pathophysiology of these symptoms be undertaken (particularly given the disconnect between their presence and disease activity in this study) and that new methods be developed to more effectively address them.

## Data availability statement

All data associated with the study described above have been incorporated into this manuscript.

## Supplementary Information

Supplementary Information.
