# Supplementary material for: Symptoms associated with healthcare resource utilization in the setting of inflammatory bowel disease
Source: Sci Rep. 2022 Jun 22;12:10577. doi: 10.1038/s41598-022-14838-y (PMC9217979; doi:10.1038/s41598-022-14838-y)
Supplement: Supplementary file 1 — Supplementary Information. [file 41598_2022_14838_MOESM1_ESM.pdf]

Symptoms Associated with Healthcare Resource Utilization in the Setting of Inflammatory Bowel Disease

Kaleb Bogale, M.D., Parth Maheshwari, M.D., Mitchell Kang, M.D., Venkata Subhash Gorrepati, M.D., M.P.H., Shannon Dalessio, M.A., Vonn Walter, Ph.D., August Stuart, M.S., Walter Koltun, M.D., Nana Bernasko, D.N.P., Andrew Tinsley, M.D., Emmanuelle D. Williams, M.D., Kofi Clarke, M.D., Matthew D. Coates, M.D., Ph.D.

Variable	Odds Ratio	95% Confidence Limits		P Value
**Abdominal Pain**	**1.99**	**1.20**	**3.21**	**0.008**
**Rectal Bleeding**	1.38	0.79	2.41	0.254
**Opioid Use**	2.35	0.93	5.92	0.071
**Steroid Use**	1.77	0.80	3.92	0.162

Supplemental Table 1. Multivariable Logistic Regression, Healthcare Resource Utilization in Crohn's Disease

Variable	Odds Ratio	95% Confidence Limits		P Value
**Age (per 1-year increase)**	1.01	0.98	1.03	0.689
**Moderate or Severe Inflammation (on endoscopic evaluation)**	1.37	0.60	3.15	0.458
**Biologic Use**	**2.62**	**1.01**	**6.82**	**0.049**
**Mesalamine Use**	0.47	0.20	1.08	0.075
**NSAID Use**	**0.19**	**0.07**	**0.54**	**0.002**
**Anxiety or Depression**	1.46	0.63	3.40	0.383
**Fatigue**	**9.48**	**2.63**	**34.23**	**<0.001**
**Abdominal Pain**	0.74	0.28	1.97	0.551

Supplemental Table 2. Multivariable Logistic Regression Model, Healthcare Resource Utilization in Ulcerative Colitis
